# VR-based path integration predicts individual risk of rapid cortical decline: a one-year longitudinal study in cognitively unimpaired adults

**DOI:** 10.1186/s13195-026-02056-x

**Published:** 2026-04-20

**Authors:** Kazuya Kawabata, Sayuri Shima, Reiko Ohdake, Epifanio Bagarinao, Yasuaki Mizutani, Harutsugu Tatebe, Riki Koike, Atsushi Kasai, Akihiro Ueda, Mizuki Ito, Junichi Hata, Shinsuke Ishigaki, Hiroshi Toyama, Takahiko Tokuda, Akihiko Takashima, Hirohisa Watanabe

**Affiliations:** 1 Department of Neurology, Fujita Health University School of Medicine, 1-98 Dengakugakubo, Kutsukake-Cho, Toyoake, Aichi 470-1192 Japan; 2 International Center for Brain Science (ICBS), Fujita Health University, Toyoake, Aichi Japan; 3 Brain and Mind Research Center, Nagoya University, Nagoya, Aichi Japan; 4 Department of Functional Brain Imaging, Institute for Quantum Medical Science, National Institutes for Quantum Science and Technology, Chiba, Japan; 5 Department of Life Science, Faculty of Science, Laboratory for Alzheimer’s Disease, Gakushuin University, Tokyo, Japan; 6 MIG (Medical Innovation Group) Inc., Tokyo, Japan; 7 Graduate School of Human Health Sciences, Tokyo Metropolitan University, Tokyo, Japan; 8 Molecular Neuroscience Research Center, Shiga University of Medical Science, Otsu, Shiga Japan; 9 Department of Radiology, Fujita Health University Hospital, Toyoake, Aichi Japan; 10 Department of Neuroetiology and Diagnostic Science, Healthy Longevity Medicine, Osaka Metropolitan University Graduate School of Medicine, Osaka, Osaka Japan

**Keywords:** Virtual reality, Navigation, Path integration, MRI, Cortical atrophy

## Abstract

**Background:**

Path integration (PI) is a navigational computation that can be affected by aging and Alzheimer’s disease (AD). Virtual-reality PI (VR-PI) preferentially engages medial temporal circuits and can reveal subtle changes that occur prior to overt impairment. Nevertheless, the longitudinal association between PI performance and structural brain changes remains unclear.

**Methods:**

In a 1-year longitudinal cohort of 71 cognitively unimpaired adults, we assessed baseline VR-PI performance (PI error and angular error), AD-related plasma biomarkers (p-tau181 and glial fibrillary acidic protein [GFAP]), and longitudinal cortical thickness and volume from MRI scans acquired one year apart, to examine whether baseline PI performance was associated with both plasma biomarkers and subsequent structural brain changes. Linear mixed-effects (LME) models were used to assess whether baseline PI performance predicted time-dependent regional thinning and atrophy.

**Results:**

Greater baseline PI error was associated with greater longitudinal cortical thinning and volume decline in the parahippocampal gyrus, middle temporal gyrus, posterior cingulate cortex, and caudal middle frontal gyrus. Similar spatial patterns were observed for angular error, indicating consistent associations across PI measures. Sensitivity analyses using extended models that additionally included either APOE ε4 status or the baseline age × time interaction, as well as analyses restricted to participants aged ≥ 40 years, did not materially alter the pattern of results. Both baseline PI error and angular error were associated with plasma p-tau181 (*r* = 0.38 for both, 95% confidence interval [CI]: 0.16–0.56), and PI error was associated with GFAP levels (*r* = 0.36, 95% CI: 0.14–0.55). Receiver operating characteristic (ROC) curve analyses showed that baseline PI error best discriminated individuals with accelerated parahippocampal thinning (10% threshold; cross-validated AUC = 0.87).

**Conclusions:**

Baseline VR-PI performance was associated with longitudinal cortical thinning and volume decline in AD-vulnerable regions, along with additional associations with plasma p-tau181 and GFAP levels. VR-PI performance may reflect both molecular (blood biomarker) and structural (MRI) signatures preceding overt clinical impairment.

**Supplementary Information:**

The online version contains supplementary material available at 10.1186/s13195-026-02056-x.

## Introduction

Spatial navigation ability decreases with age and Alzheimer’s disease (AD) progression, and impairments in path integration (PI), a core component of spatial navigation, have been implicated as an early feature of AD [1, 2]. PI can be considered primarily as the process of continuously updating the estimates of one’s position and heading by integrating self-motion cues such as optic flow, vestibular signals, and proprioception [1]. Among the brain regions supporting spatial navigation, the entorhinal cortex (EC), particularly the medial/posterior–medial EC, is vulnerable to age-related degeneration and early AD-related tau pathology. In particular, the transentorhinal/entorhinal layer II region is among the earliest sites of tau neurofibrillary tangle accumulation in AD (Braak stages I–II), as shown by classical neuropathology and recent in vivo tau PET studies [2]. The EC plays a central role in spatial coding through grid cells, which generate periodic metric representations of location that are critical for PI [3, 4]. Disruption of grid-cell function or experimental inhibition of EC neurons have been shown to impair PI in animal models, and EC dysfunction has been associated with navigational deficits in humans [5, 6]. In addition to entorhinal contributions to PI, the hippocampus plays a key role in spatial memory and goal representation during navigation [7]. Human functional neuroimaging studies using virtual reality (VR) navigation paradigms have demonstrated engagement of both the entorhinal cortex and hippocampus during PI and memory-guided navigation tasks [8, 9], supporting the use of immersive VR paradigms to probe entorhinal-dependent PI and hippocampal-dependent spatial memory.

Standard cognitive assessments, such as tests for memory and other neuropsychological characteristics, are commonly used to evaluate higher cognitive functions and to screen for cognitive impairment. Moreover, several studies have developed approaches to assess self-navigation ability using head-mounted three-dimensional (3D) VR systems [10–12]. These immersive platforms encompass the user’s visual field, allowing the assessment of EC-dependent PI errors and hippocampus-dependent spatial memory function in individuals using a VR headset. Relatedly, our previous research using a 3D VR system indicated that PI errors were associated with plasma p-tau 181, GFAP, and NfL levels even in cognitively unimpaired individuals, with significantly greater PI errors observed in APOE ε4 carriers [13]. These associations indicate that PI errors may reflect latent brain degeneration.

On the basis of the results from prior navigation studies, we hypothesized that deficits in PI performance, quantified as PI error and angular error in a VR navigation task, would predict accelerated structural changes in brain regions preferentially affected in early AD. Our primary hypothesis was that PI deficits would predict accelerated decline within an entorhinal-centered network of AD-vulnerable regions. To extend the study beyond these a priori regions, we conducted exploratory vertexwise whole-brain analyses. Specifically, we examined cortical thinning and volumetric decline using longitudinal 3D T1-weighted MRI scans acquired approximately one year apart. This approach allowed us to test whether individuals with greater PI errors showed more rapid neurodegeneration. In addition, although overt cognitive decline typically unfolds over several years, advances in high-resolution longitudinal MRI and linear mixed-effects modeling enable the detection of subtle, regionally specific structural changes over relatively short follow-up intervals of one year. To complement the longitudinal neuroimaging analyses, baseline plasma biomarker analyses of p-tau181, GFAP, and NfL were also included to examine whether previously observed cross-sectional biomarker associations with PI performance were also evident in the context of longitudinal structural brain changes. Through these analyses, we sought to identify early neuroanatomical markers of navigational decline that may precede the onset of clinical cognitive symptoms.

## Methods

### Participants

The participants were recruited between September 2021 and June 2023 in our ongoing aging registry study at Fujita Health University. Among the 111 participants included in our previously reported cross-sectional study [13], 67 completed the longitudinal follow-up. In addition, six newly included participants completed the longitudinal follow-up, resulting in a total of 73 participants with longitudinal data. After quality control at follow-up, two participants identified as p-tau181 outliers were excluded, and 71 participants were included in the final longitudinal analyses (Supplementary Figure S1). No participants were excluded after visual quality control of MRI images. All participants underwent 3-T MRI and clinical assessments of global cognitive performance using the Mini-Mental State Examination (MMSE), the Japanese version of Addenbrooke’s Cognitive Examination-Revised (ACE-R), and the Japanese version of the Montreal Cognitive Assessment (MoCA-J). Depressive symptoms were evaluated using the Geriatric Depression Scale-15 (GDS-15) as part of baseline clinical characterization. The characteristics of the participants are summarized in Table 1.

**Table 1:** Baseline characteristics

Variable	Mean ± SD or n (%)
Age, years [range]	56.5 ± 11.0 [22–79]
Sex (Female)	43 (60.6%)
Education	13.8 ± 2.2
Scan Interval, years	1.19 ± 0.25
Path Integration Error, vm	4.9 ± 2.7
Angular Error, degrees	19.4 ± 13.0
MMSE	28.9 ± 1.2
ACE-R	95.0 ± 3.6
MoCA-J	26.0 ± 2.3
GDS-15	3.0 ± 2.9
Plasma Aβ42/Aβ40	0.068 ± 0.016
Plasma GFAP, pg/mL	83.6 ± 34.7
Plasma NfL, pg/mL	11.0 ± 5.8
Plasma p-Tau181, pg/mL	1.63 ± 0.74
ApoE genotype	
ε2/ε3	4 (5.6%)
ε2/ε4	1 (1.4%)
ε3/ε3	57 (80.3%)
ε3/ε4	9 (12.7%)

*ACE-R* Addenbrooke’s Cognitive Examination-Revised, *APOE* Apolipoprotein E, *Aβ* Amyloid β, *GDS-15* Geriatric Depression Scale-15, *GFAP* Glial fibrillary acidic protein, *MMSE* Mini-Mental State Examination, *MoCA-J* Japanese version of the Montreal Cognitive Assessment, *NfL* Neurofilament light protein, *vm* virtual meter, *VR* Virtual reality

This research was approved by the Ethics Committee at Fujita Health University Hospital (approval number: HM22-407) and conformed to the Ethical Guidelines for Medical and Health Research Involving Human Subjects endorsed by the Japanese government. All participants provided written informed consent prior to joining the study and were allowed to withdraw from the study at any time.

### 3D VR navigation task

#### Apparatus

We assessed navigation performance using a head-mounted immersive 3D VR device and software provided by MIG, Inc. (Tokyo, Japan; https://www.medicalig.com), as reported previously [10, 11, 13]. The system fully encompasses the user’s visual field and simulates realistic head and body movements. The virtual environment consists of a circular arena with a diameter of 20 virtual meters (vm), bounded by walls 3 vm high. The textures were kept homogeneous, and the environment was designed free of salient distant and local landmarks to preferentially probe self-motion-based PI.

#### Procedure

Participants wore 3D VR goggles and controlled forward–backward movement using a joystick (Meta Quest 2); they physically rotated their bodies while seated on a swivel chair to turn or change headings, thereby preserving vestibular and proprioceptive cues for heading changes. Participants were first allowed to freely explore the arena with obstacles to familiarize themselves with the device and control scheme, without receiving any performance feedback.

#### Task

**Fig. 1:**
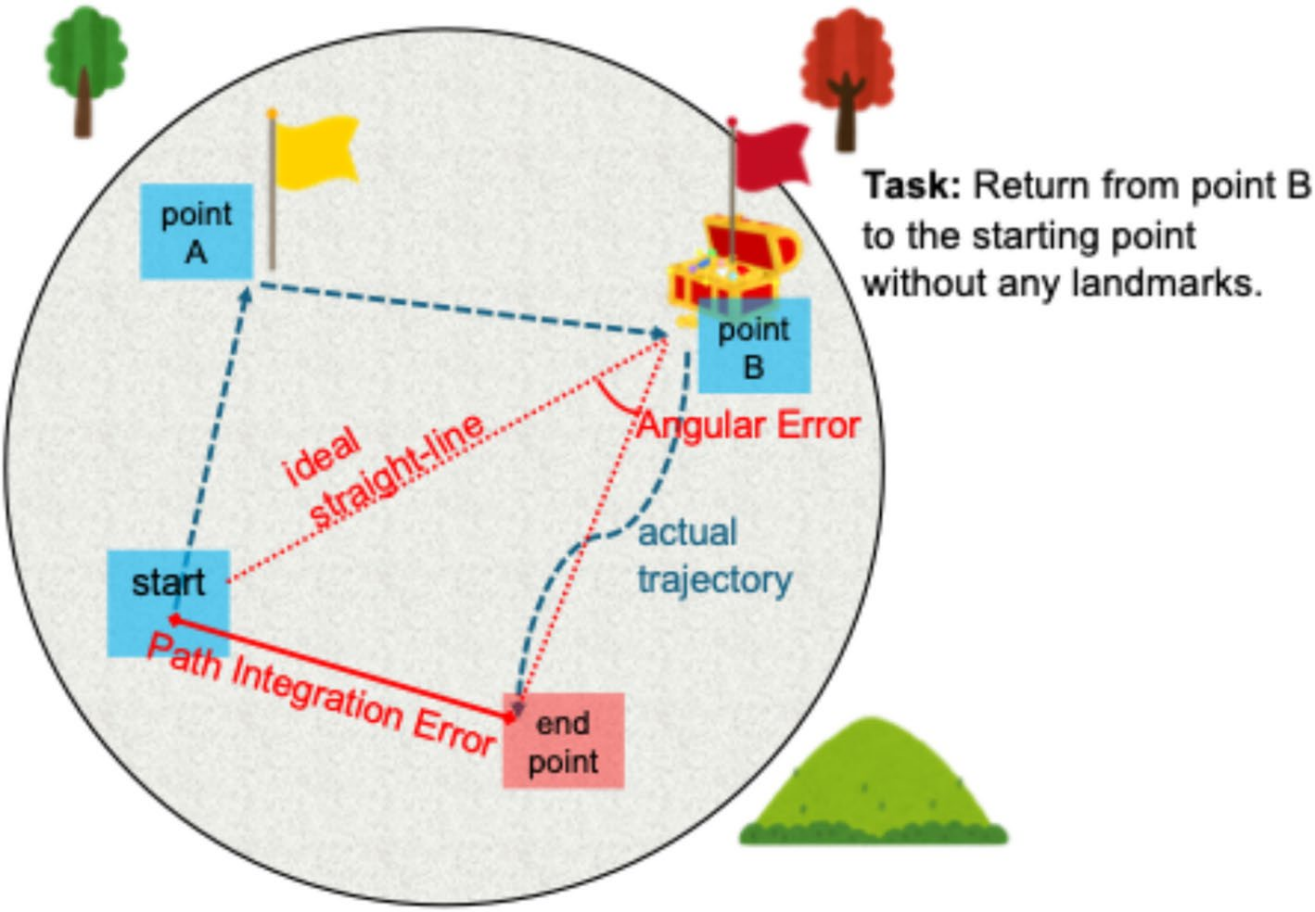
Illustration of the three-dimentional virtual reality (3D VR) task. Schematic representation of the VR path integration task. Participants navigated to Location A (yellow flag) and Location B (red flag), then returned to the starting point without visual cues. During the return phase, positions were recorded every 0.5 s to calculate (1) path integration (PI) error—the Euclidean distance between the final and starting points, and (2) angular error—the absolute angular difference between the ideal return vector and the participant’s actual return trajectory

Participants were instructed to move from the start point to Location A (yellow flag), then to Location B (red flag), and finally to return to the original starting point (Fig. 1). During the return phase, the flags that serve as landmarks were removed so that participants needed to rely on memory and internal spatial representations. Participants were given up to 30 s to return from Location B to the perceived starting point and were instructed to stop and remain at the location where they believed they had reached the original starting point. The location at which the participant stopped and remained was recorded as the final position. The participant’s x and y coordinates in virtual meters were sampled and recorded every 0.5 s.

From the recorded trajectory, we derived the following measures for each trial:

1. PI error (vm) – the Euclidean distance between the participant’s final position and the original starting point.
2. Angular error (degrees) – the absolute angular difference between the ideal return vector (straight line from Location B to the starting point) and the participant’s actual return trajectory, defined by the average direction of movement during the return phase. This metric quantifies rotational accuracy.

Each participant completed three trials, and the mean values of the PI error and angular error were used as primary behavioral measures [10, 13].

### Sample collection and assays of plasma biomarkers

Blood samples were obtained from all participants after a fasting period of more than 6 h, after which the plasma was isolated from the samples by centrifuging them for 10 min at 1,500 × g. To prevent the need for repeated freeze‒thaw cycles, the obtained plasma was aliquoted into 500 µl samples, which were promptly frozen and stored at −80 °C until analysis. The levels of plasma glial fibrillary acidic protein (GFAP), neurofilament light (NfL) protein, amyloid β (Aβ) 40, Aβ42, and p-tau181 were measured using a single-molecule array (Simoa) with the Simoa Human Neurology 4-Plex E Kit and Simoa pTau-181 V2 Advantage Kit (Quanterix, Billerica, MA, USA) according to the manufacturer’s instructions. All plasma samples were tested in duplicate. None of the participants reported recent traumatic brain injury, including concussion, within the past year.

### MRI

All participants underwent MRI scanning at Fujita Health University using a 3-T MRI scanner (Canon Medical Systems) with a 32-channel head coil. For each participant, T1-weighted images were acquired using a 3D fast field echo sequence with the following imaging parameters: repetition time (TR) = 6.6/2500 ms, echo time (TE) = 2.7 ms, flip angle (FA) = 8 degrees, field of view (FOV) = 240 × 240 mm^2^, acquisition/reconstruction matrix = 288 × 288, 230 sagittal slices with 0.8-mm slice thickness, and a voxel resolution of 0.8 × 0.8 × 0.8 mm^3^ with a total acquisition time of 5 min and 5 s. After image acquisition, MRI images were visually inspected by a neurologist to confirm adequate image quality and the absence of imaging abnormalities.

### Image preprocessing

The acquired T1-weighted MR images were preprocessed using the longitudinal preprocessing pipeline [14] in FreeSurfer version 7.4.1 (http://surfer.nmr.mgh.harvard.edu/). The analysis workflow consisted of three stages:

1. All the images were independently preprocessed using the default FreeSurfer analysis pipeline, which includes, among others, subcortical segmentation and cortical surface parcellation of the input images according to FreeSurfer’s integrated atlases.
2. An unbiased template for each participant was created using images from all time points. The generated templates were also similarly processed using the default analysis pipeline.
3. The images from all time points for each participant were registered and resampled into the unbiased participant-specific template space and then run through FreeSurfer’s analysis pipeline, but the information generated in the second stage (template processing) was used to initialize the relevant processing algorithms.

This measure was used to ensure that the images from all time points for a given participant were processed under the same initial conditions to reduce processing variability and to improve robustness and sensitivity [14]. Regional cortical volume and thickness measures, along with estimated total intracranial volume (eTIV), were obtained from longitudinally processed FreeSurfer images using the Desikan–Killiany atlas [15]. Both volume and thickness measures were analyzed since volumetric and surface-based morphometric measures provide complementary information on cortical architecture [16].

### Statistical analysis

Baseline correlations were computed using Pearson’s correlation coefficient and visualized in R (version 4.5.0). Biomarker analyses were conducted for contextual purposes to provide biological anchoring for the behavioral findings and were not included as primary predictors in the longitudinal models.

Longitudinal cortical thickness and volume changes were estimated using linear mixed-effects (LME) models fitted by maximum likelihood, as specified below, allowing estimation of within-subject change while accounting for between-subject variability in baseline levels and unequal follow-up intervals.

$$\begin{aligned} {Y}_{\mathrm{ij}}=\:&{a}_{0}+{a}_{1}{\mathrm{baselineAge}}_{\mathrm{i}}+{a}_{2}{\mathrm{Year}}_{\mathrm{ij}}+{a}_{3}{\text{PI error}}_{\mathrm{i}}+{a}_{4}{\text{PI error}}_{\mathrm{i}} \, \\&\times \, {\mathrm{Year}}_{\mathrm{ij}}+{a}_{5}{\mathrm{Sex}}_{\mathrm{i}} (+ {a}_{6}{TIV}_{\mathrm{i}})+{b}_{i0}+{b}_{i1}{\mathrm{Year}}_{\mathrm{ij}}+{\upvarepsilon }_{ij} \end{aligned}$$

$$\begin{aligned} {Y}_{\mathrm{ij}}=\:&{a}_{0}+{a}_{1}{\mathrm{baselineAge}}_{\mathrm{i}}+{a}_{2}{\mathrm{Year}}_{\mathrm{ij}}+{a}_{3}{\text{Angular error}}_{\mathrm{i}}+{a}_{4}{\text{Angular error}}_{\mathrm{i}} \, \\&\times \, {\mathrm{Year}}_{\mathrm{ij}}+{a}_{5}{\mathrm{Sex}}_{\mathrm{i}} (+ {a}_{6}{TIV}_{\mathrm{i}})+{b}_{i0}+{b}_{i1}{\mathrm{Year}}_{\mathrm{ij}}+{\upvarepsilon }_{ij} \end{aligned}$$

In the above equations, *Y*_*ij*_ represents either cortical thickness or regional volume for the *i*_th_ participant at scan *j*. The *a* variables are the coefficients associated with the fixed-effect terms (i.e., effects common to all participants), the terms *b*_*i0*_ and *b*_*i1*_ represent participant-specific random intercepts and slopes, and *ε*_*ij*_ is the residual error term. Fixed-effect covariates included baseline age, year from baseline, sex, and (for the volume model) total intracranial volume (TIV). *PI error*_*i*_ and *Angular error*_*i*_ represent navigation error metrics defined as the mean PI error or the mean angular error at the baseline. These two metrics were modeled separately. The interaction terms *PI error*_*i*_ × *Year*_*ij*_ and *Angular error*_*i*_ × *Year*_*ij*_ represent whether baseline navigation performance is associated with longitudinal changes in cortical thickness or volume. All fixed-effect coefficients are presented as standardized estimates. We used the *fitlme* function in MATLAB (R2024a, MathWorks, Natick, MA, USA) for LME analyses. For multiple comparisons, we applied false discovery rate (FDR) correction using the Benjamini‒Hochberg procedure and considered an FDR-corrected q < 0.05 to indicate statistical significance.

To assess robustness, we performed sensitivity analyses using the following extended models and compared them with the base model. First, the models were refitted after the inclusion of APOE ε4 carrier status and its interaction with time (APOE ε4 carrier status and APOE ε4 carrier status × Year). Second, the models were refitted after the interaction of baseline age and year (baselineAge × Year) was added as a term. Model fit between the original and extended models was assessed using Akaike’s information criterion (AIC) and likelihood ratio test (LRT). Details of the comparisons are provided in Supplementary Table S3. Furthermore, we conducted an additional sensitivity analysis using the original models restricted participants aged 40 years or older.

For the subanalysis, we explored whether participants could be classified according to greater thinning or volume decline in the parahippocampal gyrus and posterior cingulate cortex, regions that showed significant effects in the LME analysis and are known to be vulnerable to AD–related neurodegeneration. In these ROC analyses, baseline PI error served as the predictor variable. Annual symmetrized percent change (SPC) values (%/year) were calculated to quantify longitudinal changes. Participants were then classified into the bottom 10% (*n* = 8), 15% (*n* = 11), and 20% (*n* = 15) of the annual SPC distribution, representing the greatest decline, for each region of interest. Receiver operating characteristic (ROC) analyses were subsequently performed to evaluate the discriminative ability of baseline PI measures for identifying individuals within these subgroups. The Youden index was applied to determine the optimal cutoff, and the sensitivity, specificity, and accuracy at this threshold were reported with 95% confidence intervals (95% CI) estimated by nonparametric bootstrap resampling with 5000 iterations. To derive more robust estimates of generalization performance, repeated stratified fivefold cross-validation was conducted, and performance metrics were aggregated across folds and repeats. This analysis was conducted using R.

## Results

### Demographics and characteristics

The demographics and characteristics of the participants are shown in Table 1 (*N* = 71). The mean age was 56.5 years (SD = 11.0), with participants ranged in age from 22 to 79 years. The age distribution by decade was as follows: 20–29 years (*n* = 1), 30–39 years (*n* = 2), 40–49 years (*n* = 16), 50–59 years (*n* = 27), 60–69 years (*n* = 16), and ≥ 70 years (*n* = 9). The mean follow-up period was 1.19 years (SD = 0.25), the mean PI error distance was 4.9 vm (SD = 2.7), and the mean angular error was 19.4 degrees (SD = 13.0). Both PI error and angular error distributions showed a single dominant peak, with right-skewed tails without clustering near the lower bound or saturation at the upper end, as shown in the density plots in Supplementary Figure S2.

**Fig. 2:**
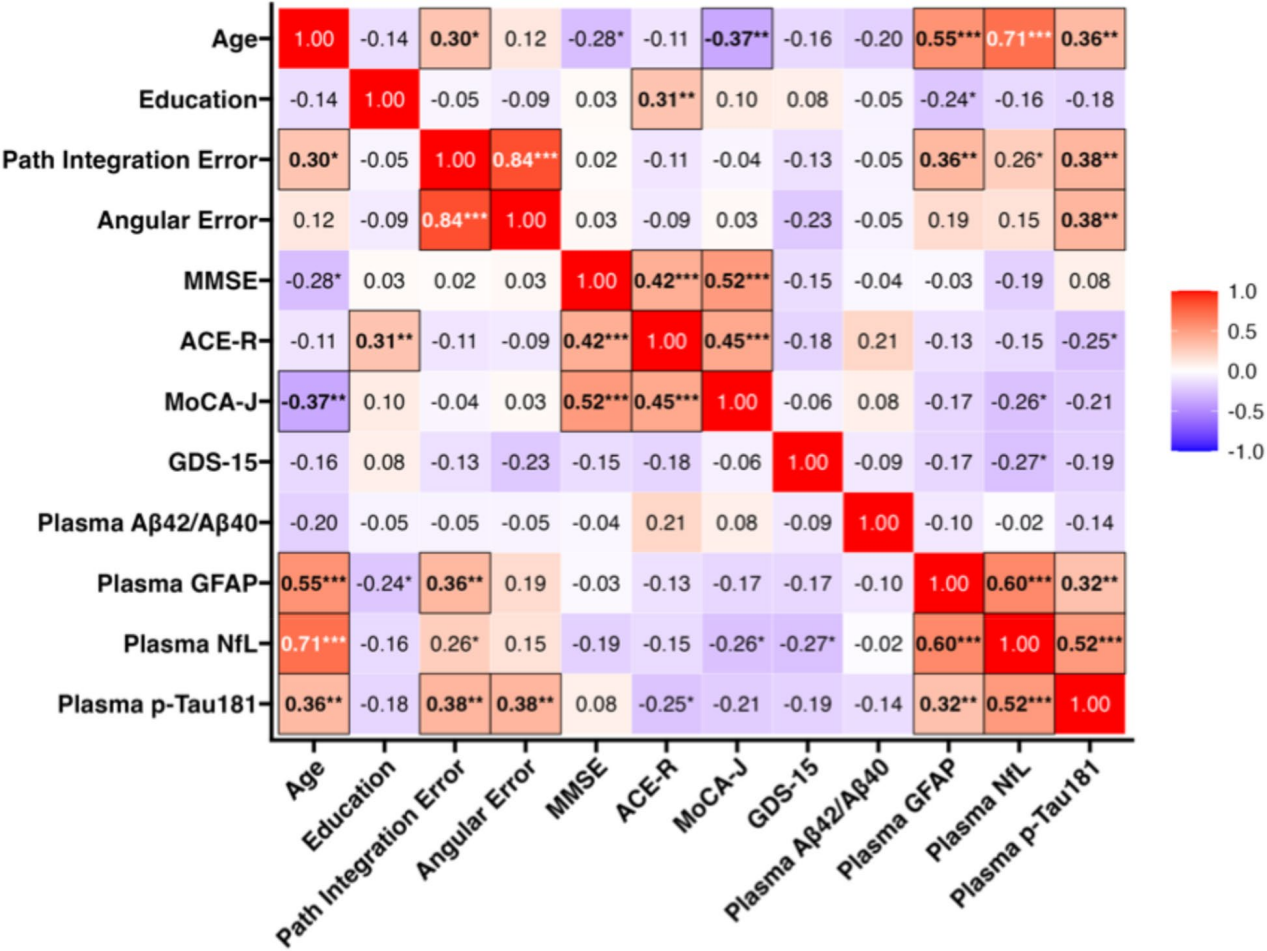
Correlation among baseline demographic, behavioral, and biomarker measures. The heatmap shows the Pearson’s correlation coefficients between variables. Numerical values within each cell represent correlation coefficients. Asterisks denote nominal statistical significance (*p < 0.05, **p < 0.01, ***p < 0.001). Correlations that remained significant after false discovery rate (FDR) correction (q < 0.05) are indicated by bold text and black outlines. Aβ, amyloid β; ACE-R, Addenbrooke’s Cognitive Examination-Revised; GDS-15, Geriatric Depression Scale-15; GFAP, glial fibrillary acidic protein; MoCA-J, Japanese version of the Montreal Cognitive Assessment; NfL, neurofilament light protein

A correlation matrix of the behavioral data is shown in Fig. 2. Pearson correlation analysis revealed a strong association between PI error and angular error (*r* = 0.84; 95% CI, 0.76–0.90; *p* < 0.001; FDR-corrected q < 0.001). PI error was modestly correlated with age (*r* = 0.30; 95% CI, 0.07–0.50; *p* = 0.010; q = 0.043), plasma GFAP (*r* = 0.36; 95% CI, 0.14–0.55; *p* = 0.002; q = 0.010) and p-tau181 (*r* = 0.38; 95% CI, 0.16–0.56; *p* = 0.001; q = 0.008). Angular error was correlated with p-tau181 (*r* = 0.38; 95% CI, 0.16–0.56; *p* = 0.001; q = 0.008) but not with age. Correlation matrices adjusted for age and sex, as well as age, sex, and storage time for biomarkers are shown in Supplementary Figure S3. Angular error remained significant with p-tau181 (*r* = 0.36; 95% CI, 0.14–0.55; *p* = 0.002; q = 0.015), while PI error was not significantly correlated with p-tau181 after correcting multiple comparisons (*r* = 0.30; 95% CI, 0.07–0.50; *p* = 0.010; q = 0.062).

### PI error: longitudinal cortical thickness and volume changes

In the LME model for cortical thickness, the main effect of baseline PI error (representing cross-sectional differences at baseline) was not statistically significant. In contrast, the PI error × Year interaction was significantly associated with greater longitudinal cortical thinning over one year in multiple cortical regions with larger baseline PI errors. Regions with significant thickness effects included the banks of the left superior temporal sulcus (standardized coefficient a_4_ = −0.142), the left middle temporal gyrus (a_4_ = −0.219), the left parahippocampal gyrus (a_4_ = −0.155), the left superior temporal gyrus (a_4_ = −0.212), the left supramarginal gyrus (a_4_ = −0.201), the left temporal pole (a_4_ = −0.270), the left transverse temporal gyrus (a_4_ = −0.192), the right caudal middle frontal gyrus (a_4_ = −0.203), and the right superior temporal gyrus (a_4_ = −0.166). A positive association was observed in the left frontal pole (a_4_ = 0.226).

In the LME volume analysis, baseline mean PI error itself showed no significant main effects, whereas the PI error × Year interaction was associated with greater longitudinal volume decline in the left caudal anterior cingulate cortex (a_4_ = −0.052), left fusiform gyrus (a_4_ = −0.081), left middle temporal gyrus (a_4_ = −0.066), left parahippocampal gyrus (a_4_ = −0.108), bilateral posterior cingulate cortex (left: a_4_ = −0.053; right: a_4_ = −0.047), right caudal middle frontal gyrus (a_4_ = −0.069), and right lateral orbitofrontal cortex (a_4_ = −0.120). Among these regions, a significant main effect of sex was observed only for volume in the right lateral orbitofrontal cortex after FDR correction (q < 0.05), whereas no other regions showed significant sex effects.

**Fig. 3:**
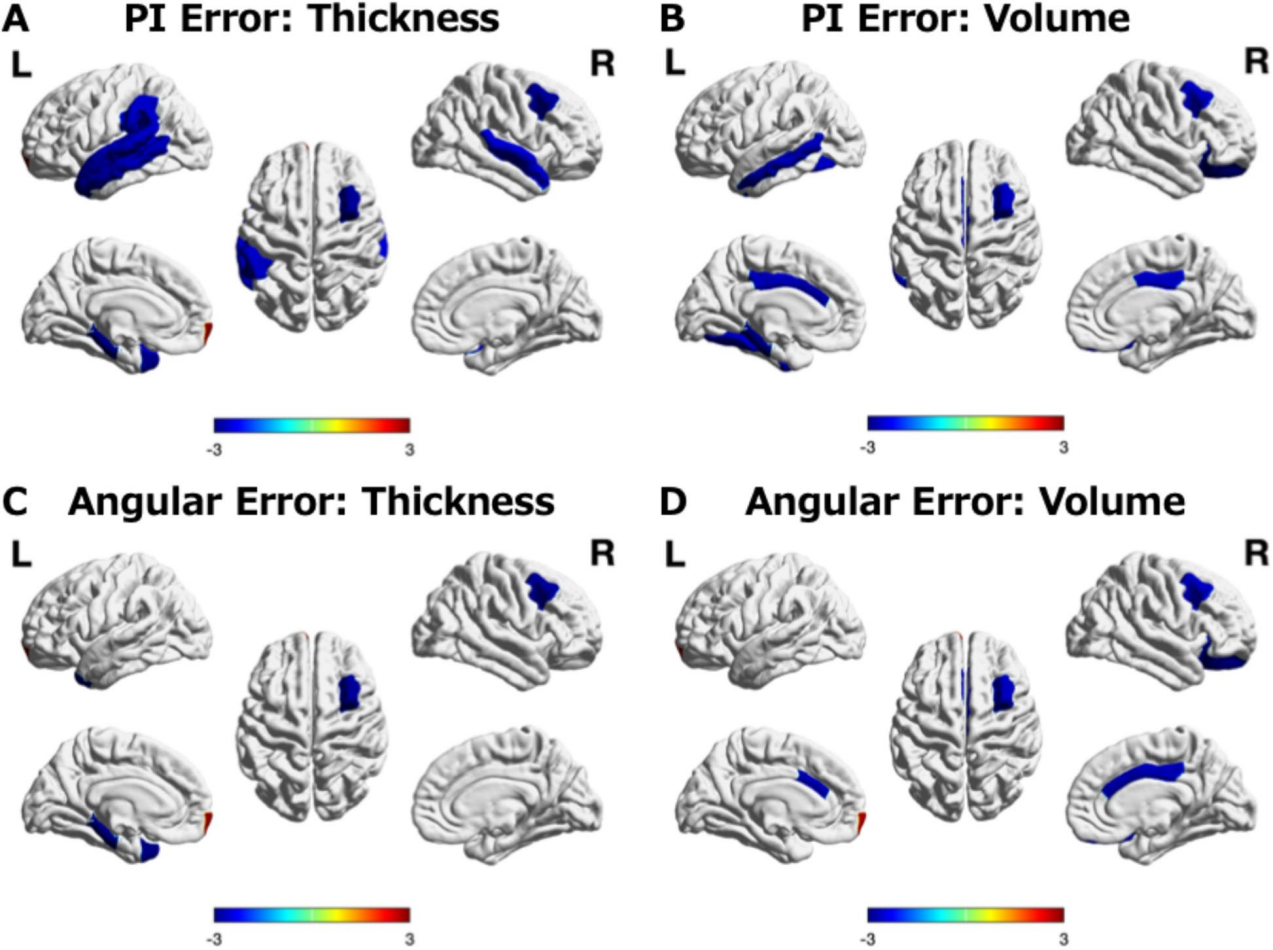
Time-dependent thickness and volume changes associated with 3D VR navigation. Cortical surface maps showing regions with longitudinal changes associated with PI performance (PI error and Angular error). Blue indicates greater cortical volume or thickness decline, whereas red indicates relatively preserved regions. Only regions surviving false discovery rate (FDR) correction (q < 0.05) are displayed, applied separately to the PI error × Year and Angular error × Year interaction terms in their respective linear mixed-effects models. Colors represent the corresponding t values, with the color scale truncated at − 3 to + 3; values beyond these limits are shown at the extremes of the color bar. The cortex was parcellated into 34 regions of interest (ROIs) per hemisphere according to the Desikan–Killiany atlas. **A** Thickness changes associated with PI error. **B** Volume changes associated with PI error. **C** Thickness changes associated with angular error. **D** Volume changes associated with angular error

Significant regions are shown in Fig. 3 (3A for thickness and 3B for volume), and the corresponding standardized coefficients for all fixed effects, including adjustment covariates, are summarized in Supplementary Table S1.

### Angular error: longitudinal cortical thickness and volume changes

In the LME model for cortical thickness, the main effect of baseline angular error was not statistically significant. However, the interaction term Angular error × Year was associated with accelerated thinning in several regions. Regions with significant negative thickness effects included the left parahippocampal gyrus (a_4_ = −0.125), left temporal pole (a_4_ = −0.232), and right caudal middle frontal gyrus (a_4_ = −0.181). A positive association was observed in the left frontal pole (a_4_ = 0.181). The regions where the effects survived correction are shown in Fig. 3 (3C for thickness and 3D for volume), and the corresponding standardized coefficients for all fixed effects, including adjustment covariates, are also summarized in Supplementary Table S1.

In the volume analysis, baseline mean angular error did not show significant main effects, but the Angular error × Year interaction predicted accelerated volume loss in the bilateral caudal anterior cingulate gyrus (left, a_4_ = −0.045; right, a_4_ = −0.044), right caudal middle frontal gyrus (a_4_ = −0.062), right lateral orbitofrontal gyrus (a_4_ = −0.098), and right posterior cingulate gyrus (a_4_ = −0.045). A positive association was observed in the left frontal pole (a_4_ = 0.091).

### Additional sensitivity analyses: extended models with APOE ε4 carrier status and the APOE ε4 × Year (time) interaction term, and the baseline age × Year interaction term

A summary of the regions that are significant in the extended models is provided in Supplementary Figures S4 and S5, and Table S2. For cortical thickness, the left parahippocampal gyrus, left temporal pole, right caudal middle frontal gyrus, and left frontal pole remained consistently significant for either the PI error × Year or the Angular error × Year interaction across all original and extended models. For regional volumes, the PI error models consistently identified the involvement of the left caudal anterior cingulate cortex, parahippocampal gyrus, posterior cingulate cortex, and right orbitofrontal gyrus, whereas the angular error models consistently implicated the left frontal pole, caudal anterior cingulate cortex, caudal middle frontal gyrus, lateral orbitofrontal cortex, and posterior cingulate cortex.

According to the model comparison results, adding APOE ε4 status and its interaction with Year or the baseline Age × Year interaction generally provided limited additional explanatory value beyond that of the base models (Supplementary Table S3). Specifically, in the PI error models, no regional cortical thickness measurements showed significant improvement with either covariate. Regarding volume, only the left parahippocampal gyrus showed a significant improvement when APOE ε4 status was added (LRT *p* = 0.029). In angular error models, adding APOE ε4 improved model fit for the right posterior cingulate cortex in thickness analyses (*p* = 0.022) and for the right rostral anterior cingulate cortex in volume analyses (*p* = 0.014). No regions demonstrated significant improvement when the baseline Age × Year interaction was added. Across both error types and modalities, adding APOE ε4 or Age × Year did not improve model fit, as most regions had higher AIC values in the extended models than in the base models (ΔAIC > 0). The set of model comparison statistics (comprising AIC, ΔAIC, LRT χ^2^, degrees of freedom, and p values) is reported in Supplementary Tables S3.

### Additional sensitivity analysis restricted to participants aged ≥ 40 years

When restricted to participants aged ≥ 40 years (*n* = 68), the overall pattern of results remained materially unchanged. A summary of the significant regions is provided in Supplementary Figure S6 and Table S2. For cortical thickness, the left parahippocampal gyrus, left temporal pole, right caudal middle frontal gyrus, and left frontal pole remained significant for either the PI error × Year or the Angular error × Year interaction. For regional volumes, the PI error models similarly identified the left caudal anterior cingulate cortex, fusiform gyrus, middle temporal gyrus, parahippocampal gyrus, right caudal middle frontal gyrus, and lateral orbitofrontal gyrus. The angular error models showed a pattern consistent with the original analyses, including the left frontal pole, right caudal anterior cingulate cortex, caudal middle frontal gyrus, lateral orbitofrontal cortex, and posterior cingulate cortex. A small number of additional regions (left inferior parietal cortex and insula) were also observed in the restricted analysis, as shown in Supplementary Figure S6 and Table S2.

### ROC analysis for classifying individuals with greater thinning and volume decline in the parahippocampal gyrus and posterior cingulate cortex

**Fig. 4:**
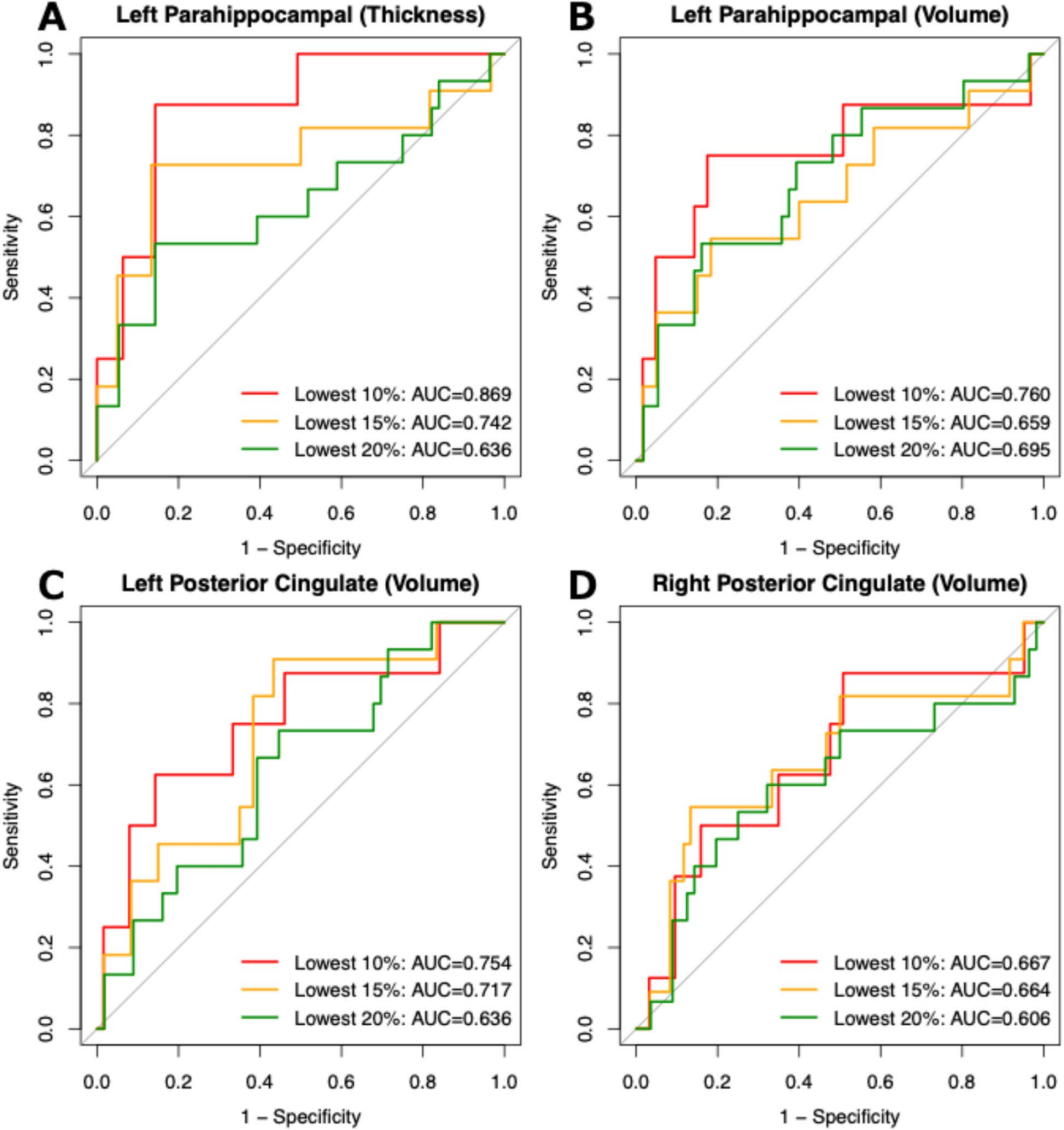
ROC analysis of thickness and volume measures to distinguish individuals with greater decline based on PI error. Participants were classified into the bottom 10%, 15%, and 20% of the annual symmetrized percent change distribution for each region of interest (ROI), representing the greatest decline. Receiver operating characteristic (ROC) analyses were conducted to test whether PI error could distinguish individuals with steeper cortical thinning or volume loss in each ROI from others. The analyses focused on regions that showed significant associations in the linear mixed-effects model, specifically the cortical thickness and volume of the left parahippocampal gyrus and the volumes of the left and right posterior cingulate gyri, which are key nodes of the default mode network and vulnerable to Alzheimer’s disease. **A **ROC curves for left parahippocampal cortical thickness. **B** ROC curves for left parahippocampal volume. **C** ROC curves for left posterior cingulate volume. **D** ROC curves for right posterior cingulate volume

We evaluated whether baseline PI error discriminated participants who subsequently exhibited an accelerated decline in target regions identified by the longitudinal analyses. Specifically, using baseline PI error as the predictor in ROC analyses, we examined changes in cortical thickness and volume of the left parahippocampal gyrus, and volumes of the left and right posterior cingulate cortices, defining cases as participants in the bottom 10%, 15%, or 20% of the annual SPC distribution for each region. The ROC curve results are summarized in Fig. 4 and Supplementary Table S4. Baseline PI error showed the best discrimination for participants with accelerated parahippocampal thinning; at the 10% threshold (*n* = 8 of 71 participants), the cross-validated AUC was 0.87 (95% CI: 0.74–0.97). At the cutoff identified by the Youden index, the sensitivity was 0.88 (95% CI: 0.67–1.00), and the specificity was 0.86 (95% CI: 0.53–0.95); the mean cross-validated accuracy across folds was 0.84. For parahippocampal volume, PI error showed moderate discrimination (AUC up to 0.76), whereas discrimination was lower for posterior cingulate volume, although it was still informative (AUC range 0.61–0.75). Given the small number of participants in the subgroups, the confidence intervals were wide. In addition, these subgroup-based classification analyses should be interpreted cautiously, as they may be sensitive to sampling variability and potential overfitting.

## Discussion

In this longitudinal MRI study of cognitively unimpaired adults, greater baseline PI error was associated with accelerated cortical thinning and volume loss over one year across multiple brain regions. Specifically, greater PI error predicted a steeper decline in cortical thickness in the left temporal cortex, including the parahippocampal gyrus, and in the right caudal middle frontal and superior temporal gyri. Volume analyses revealed concordant associations in the parahippocampal gyrus, bilateral posterior cingulate cortex, left caudal anterior cingulate gyrus, left fusiform gyrus, left middle temporal gyrus, right caudal middle frontal gyrus, and right lateral orbitofrontal gyrus. Although baseline angular error was strongly correlated with PI error, angular error showed a weaker association with age and displayed a partially overlapping spatial pattern of longitudinal decline. Sensitivity analyses using extended models that included APOE ε4 status × Year or baseline Age × Year interactions, as well as analyses restricted to participants aged 40 years or older, produced results consistent with those of the primary models. Taken together, the results reflect convergent effects across thickness and volume measures in the parahippocampal gyrus, caudal middle frontal gyrus, and middle temporal gyrus, suggesting robust longitudinal vulnerability of an entorhinal cortex–centered navigation network and connected hubs.

### Greater cortical thickness and volume decline in individuals with greater PI errors

The parahippocampal gyrus is a core node in the medial–temporal navigation circuitry [17, 18] and, together with the hippocampus and retrosplenial cortex, contributes to encoding translational and rotational self-motion signals during the PI [19]. The involvement of the caudal middle frontal gyrus, a part of the dorsolateral prefrontal cortex, is consistent with a frontal control contribution (e.g., goal maintenance and trajectory updating) that complements medial temporal computations for visuospatial planning [20]. Both the parahippocampal and caudal middle frontal regions were also associated with angular error. Furthermore, ROC analysis revealed that baseline PI error best discriminated individuals who subsequently exhibited accelerated parahippocampal thinning (cross-validated AUC = 0.87 at the 10% SPC threshold). Cortical thickness and regional volume capture partially distinct aspects of cortical morphology, and thickness-based measures may be more sensitive to subtle cortical alterations than volumetric measures in the present study [16]. Taken together, these findings suggest that greater PI error with parahippocampal thinning may indicate early neuroanatomical vulnerability; however, this interpretation requires replication and validation.

Lateral temporal areas, including the motion-sensitive human middle temporal complex **(**hMT+), are also involved in the optic-flow and heading processing that underpins egocentric self-motion estimation; structural alterations here could therefore reduce the fidelity of self-motion cues and contribute to PI errors [8, 21], but direct confirmation would require task-based fMRI, effective connectivity analyses, or causally oriented studies.

The posterior cingulate cortex emerged repeatedly in the volume analyses. As a central hub of the default-mode network with dense connections to the medial–temporal and frontal cortices, the posterior cingulate cortex integrates memory- and control-related signals [22]. Atrophy and hypometabolism of the posterior cingulate cortex have been reported even in preclinical AD [23, 24]. Furthermore, prior studies showing reduced functional connectivity suggest that structural decline in the posterior cingulate cortex may indirectly contribute to impaired navigation by disrupting large-scale memory and control networks, highlighting impaired PI performance as a potential early marker of neurodegeneration [25].

Importantly, the structural decline observed in this study does not, by itself, demonstrate Alzheimer’s disease pathology. Although several affected regions overlap with areas implicated early in AD (for example, regions commonly assigned to Thal phase 1) [26], we observed no convincing evidence of entorhinal cortical atrophy in our atlas-based analyses, nor evidence of amyloid positivity in this cohort. Thus, we do not interpret our findings as evidence of amyloid pathology per se. Rather, PI-related thinning in the medial temporal and temporoparietal cortices likely indicates vulnerability within navigation and memory networks that may reflect a mixture of normative aging, vascular or other non-AD processes, and early tau-related changes supported in our sample by correlations with plasma p-tau181. Our longitudinal whole-brain analyses were based on a deliberately conservative and mechanism-driven hypothesis: we tested whether baseline PI errors mark network susceptibility that precedes more overt structural decline, with subsequent thinning and volume loss arising in connected regions such as the parahippocampal gyrus and posterior cingulate cortex, irrespective of amyloid status. This interpretation remains testable by stratifying future samples with amyloid/tau PET and next-generation plasma assays (e.g., for p-tau217).

### Associations among PI error, angular error and aging

At baseline, angular error was strongly correlated with PI error, corresponding to regionally consistent patterns of greater cortical thickness and volume decline. Both were associated with plasma p-tau181, whereas angular error showed a weaker association with age than PI error did.

A progressive decline in navigational ability with age has been demonstrated in both rodents and humans [1]. Because spatial navigation is a multifaceted cognitive operation, impairments may emerge at multiple processing stages. Older adults show increased errors in VR-based PI tasks comparable to those observed in real-world navigation tasks performed under sensory-restricted conditions [27]. Successful PI depends on the accurate integration of linear and angular self-motion cues [28]. The strong correlation between angular error and PI error suggests that angular error is a major contributor to degraded PI performance. In support of this relationship, computational and behavioral studies have reported that overestimation of turning angles is a major driver of PI errors in individuals with mild cognitive impairment (MCI), particularly in those with biomarkers consistent with early AD [28]. In middle-aged participants stratified by hereditary and physiological AD risk, PI impairments emerge prior to measurable deficits in other spatial behaviors or episodic memory [29]. Longitudinal work in individuals with MCI has further shown that both the PI and angular errors predict 12-month cognitive decline [10]. Complementing these findings, Colmant et al. reported that poor angular estimation may serve as an early cognitive marker of tau pathology, whereas distance estimation errors appear to be more strongly linked to normative aging than to AD [30]. The relatively weak association between chronological age and angular error should be verified in independent cohorts; however, if the association is confirmed, angular error could represent a behavioral index that is comparatively unsusceptible to age confounding and sensitive to early pathological change.

### Limitations

This study has several limitations. First, cortical thickness and volumetric estimates were derived from the FreeSurfer longitudinal pipelines, which, while reducing variability across time points, may still introduce within-individual variability. Second, although our 3D VR navigation system demonstrated potential for detecting early PI deficits, it may not fully capture the same processes that occur during real-world PI. While VR provides rotational vestibular input through head movement, it lacks the linear acceleration cues and proprioceptive feedback associated with actual walking, resulting in incomplete replication of real-world sensory integration. Nevertheless, the VR system offers a practical and controlled approach for assessing PI function. Third, although PI error is thought to reflect functional alterations within the entorhinal areas, this study did not demonstrate direct associations between PI error and greater cortical thickness or volume decline within the entorhinal cortex. This may reflect limited sensitivity of conventional MRI-based thickness and volumetric measures to detect early entorhinal changes in cognitively unimpaired individuals. Fourth, the observed left-lateralization patterns and the relatively preserved thickness and volume of the frontal pole require replication in larger samples and diverse populations. Although spatial navigation is often considered to preferentially engage right-hemisphere networks, prior human studies have also demonstrated left-hemisphere involvement during PI and memory-guided navigation [31, 32]. The underlying reasons for the relative preservation of the frontal pole, whether they reflect compensatory or reserve-related mechanisms, remain unclear. In addition, given that the present cohort comprised cognitively unimpaired individuals, inter-individual heterogeneity is likely, which may have contributed to the observed laterality. Finally, because navigation strategies may differ across cultural and educational backgrounds, the generalizability of these findings may be limited. Future studies incorporating advanced EC segmentation methods and larger, more diverse longitudinal cohorts are needed to clarify whether structural alterations within the EC underlie the PI impairments observed during aging and early neurodegenerative processes, including preclinical AD.

## Conclusions

The results of this longitudinal study suggest that greater PI error is associated with greater cortical thinning and volume loss in several regions, such as the parahippocampal gyrus and posterior cingulate cortex, even among individuals who remain cognitively unimpaired. These findings indicate that PI performance may reflect subtle neuroanatomical vulnerability and PI-associated network susceptibility, along with additional associations with plasma biomarkers. This overall pattern remained largely consistent across sensitivity analyses accounting for APOE ε4 status, baseline age × time interaction, and restriction to participants aged 40 years or older. Overall, PI performance may serve as an informative marker of early neuroanatomical susceptibility, with potential relevance to AD-related vulnerability, although further work is needed to determine whether PI impairment can identify individuals at elevated risk for clinical decline at relatively early stages.

## Supplementary Information

Supplementary Material 1.

## Data Availability

The data supporting the conclusions of this article will be made available by the corresponding author upon reasonable request.
