# Supplementary material for: VR-based path integration predicts individual risk of rapid cortical decline: a one-year longitudinal study in cognitively unimpaired adults
Source: Alzheimers Res Ther. 2026 Apr 20;18:134. doi: 10.1186/s13195-026-02056-x (PMC13227782; doi:10.1186/s13195-026-02056-x)
Supplement: Supplementary file 1 — Supplementary Material 1. [file 13195_2026_2056_MOESM1_ESM.docx]

**Supplementary Figure S1. Participant flow for longitudinal analyses**

**Supplementary Figure S2. Kernel density plots of navigation performance measures**

The left panel shows the distribution of path integration (PI) error, and the right panel shows the distribution of angular error. The x-axis represents the mean PI error distance (virtual meters, vm) and the mean angular error (degrees), respectively, averaged across three trials per participant.

**Supplementary Figure S3. Adjusted correlations among baseline demographic, behavioral, and biomarker measures**

The heatmap shows Pearson’s correlation coefficients between variables. Colors indicate the strength and direction of correlations (blue to red). Numerical values within each cell represent correlation coefficients. Asterisks denote nominal statistical significance (*p < 0.05, **p < 0.01, ***p < 0.001). Correlations that remained significant after false discovery rate (FDR) correction (q < 0.05) are indicated by bold text and black outlines.

The left panel shows correlations adjusted for age and sex for all variables, with additional adjustment for biomarker storage duration applied only to plasma biomarkers, whereas the right panel shows correlations adjusted for age and sex.

**Supplementary Figure S4. Time-dependent cortical thickness and volume changes associated with 3D VR navigation, adjusted for APOE ε4 status and its interaction with time**

Regions in blue represent significantly faster cortical volume or thickness decline associated with higher PI error or Angular error, whereas red indicates relatively preserved areas in the extended model. These effects were identified at a significance level of q < 0.05 after correction for multiple comparisons using the false discovery rate (FDR), applied separately to the PI error × Year and Angular error × Year interaction terms in their respective linear mixed-effects models. The color bar (blue to red) represents t values of the significant regions, with the color scale truncated at −3 to +3; values beyond these limits are shown at the extremes of the color bar.

A. Thickness, correlation with PI error; B. Volume, correlation with PI error; C. Thickness, correlation with angular error; D. Volume, correlation with angular error.

**Supplementary Figure S5. Time-dependent cortical thickness and volume changes associated with 3D VR navigation, adjusted for the interaction between baseline age and time**

Regions in blue represent significantly faster cortical volume or thickness decline associated with higher PI error or angular error, whereas red indicates relatively preserved areas in the extended model. These effects were identified at a significance level of q < 0.05 after correction for multiple comparisons using the false discovery rate (FDR), applied separately to the PI error × Year and Angular error × Year interaction terms in their respective linear mixed-effects models. The color bar (blue to red) represents t values of the significant regions, with the color scale truncated at −3 to +3; values beyond these limits are shown at the extremes of the color bar.

A. Thickness, correlation with PI error; B. Volume, correlation with PI error; C. Thickness, correlation with angular error; D. Volume, correlation with angular error.

**Supplementary Figure S6. Time-dependent cortical thickness and volume changes associated with 3D VR navigation in aged 40 years or older**

Regions in blue represent significantly faster cortical volume or thickness decline associated with higher PI error or angular error, whereas red indicates relatively preserved areas in the extended model. These effects were identified at a significance level of q < 0.05 after correction for multiple comparisons using the false discovery rate (FDR), applied separately to the PI error × Year and Angular error × Year interaction terms in their respective linear mixed-effects models. The color bar (blue to red) represents t values of the significant regions, with the color scale truncated at −3 to +3; values beyond these limits are shown at the extremes of the color bar.

A. Thickness, correlation with PI error; B. Volume, correlation with PI error; C. Thickness, correlation with angular error; D. Volume, correlation with angular error.

**Supplementary Table S1. Standardized coefficients from linear mixed-effects models**

| **PI Error, Thickness** | **baseline Age** | **Year** | **PI error** | **PI error × Year** | **Sex** |  |
| --- | --- | --- | --- | --- | --- | --- |
| Lt banks of the superior temporal sulcus | -0.297 | 0.087 | -0.058 | -0.142 | 0.083 |  |
| Lt middle temporal gyrus | -0.406 | 0.211 | -0.033 | -0.219 | -0.041 |  |
| Lt parahippocampal gyrus | 0.043 | 0.118 | -0.092 | -0.155 | -0.222 |  |
| Lt superior temporal gyrus | -0.424 | 0.144 | 0.046 | -0.212 | 0.005 |  |
| Lt supramarginal gyrus | -0.457 | 0.147 | -0.049 | -0.201 | -0.068 |  |
| Lt frontal pole | 0.021 | -0.132 | -0.152 | 0.226 | -0.188 |  |
| Lt temporal pole | -0.223 | 0.218 | 0.172 | -0.270 | 0.012 |  |
| Lt transverse temporal gyrus | -0.026 | 0.120 | 0.100 | -0.192 | 0.110 |  |
| Rt caudal middle frontal gyrus | -0.287 | 0.106 | -0.094 | -0.203 | 0.173 |  |
| Rt superior temporal gyrus | -0.402 | 0.059 | 0.117 | -0.166 | 0.033 |  |
| **PI Error, Volume** | **baseline Age** | **Year** | **PI error** | **PI error × Year** | **Sex** | **eTIV** |
| Lt caudal anterior cingulate cortex | -0.063 | 0.022 | 0.070 | -0.052 | -0.264 | 0.377 |
| Lt fusiform gyrus | -0.392 | 0.055 | 0.069 | -0.081 | 0.204 | 0.266 |
| Lt middle temporal gyrus | -0.272 | 0.052 | -0.111 | -0.066 | -0.167 | 0.687 |
| Lt parahippocampal gyrus | -0.127 | 0.080 | -0.052 | -0.108 | 0.050 | 0.248 |
| Lt posterior cingulate cortex | -0.202 | 0.011 | 0.043 | -0.053 | 0.066 | 0.422 |
| Rt caudal middle frontal gyrus | -0.270 | 0.030 | 0.061 | -0.069 | 0.047 | 0.550 |
| Rt lateral orbitofrontal cortex | -0.273 | 0.081 | 0.020 | -0.120 | 0.313 | 0.386 |
| Rt posterior cingulate cortex | -0.286 | 0.009 | 0.036 | -0.047 | 0.077 | 0.522 |
| **Angular Error, Thickness** | **baseline Age** | **Year** | **Angular error** | **Angular error × Year** | **Sex** |  |
| Lt parahippocampal gyrus | 0.022 | 0.087 | -0.081 | -0.125 | -0.218 |  |
| Lt frontal pole | -0.001 | -0.086 | -0.088 | 0.181 | -0.175 |  |
| Lt temporal pole | -0.218 | 0.177 | 0.192 | -0.232 | 0.013 |  |
| Rt caudal middle frontal gyrus | -0.316 | 0.078 | -0.114 | -0.181 | 0.177 |  |
| **Angular Error, Volume** | **baseline Age** | **Year** | **Angular error** | **Angular error × Year** | **Sex** | **eTIV** |
| Lt caudal anterior cingulate cortex | -0.060 | 0.013 | 0.070 | -0.045 | -0.264 | 0.379 |
| Lt frontal pole | -0.080 | -0.073 | -0.068 | 0.091 | 0.011 | 0.002 |
| Rt caudal anterior cingulate cortex | -0.191 | 0.011 | 0.072 | -0.044 | -0.019 | 0.349 |
| Rt caudal middle frontal gyrus | -0.257 | 0.021 | 0.044 | -0.062 | 0.044 | 0.547 |
| Rt lateral orbitofrontal cortex | -0.273 | 0.063 | -0.003 | -0.098 | 0.307 | 0.387 |
| Rt posterior cingulate cortex | -0.271 | 0.005 | -0.030 | -0.045 | 0.065 | 0.523 |

Standardized fixed-effect beta coefficients were derived from the original linear mixed-effects models. Sex was included as a categorical variable (reference category: female).

**Supplementary Table S2. Significant brain regions identified across linear mixed-effects models.**

|  | PI Error | | | | Angular Error | | | |
| --- | --- | --- | --- | --- | --- | --- | --- | --- |
| Thickness | Original | +APOE ε4 | +bAge × Year interaction | Age ≥40 | Original | +APOE ε4 | +bAge × Year interaction | Age ≥40 |
| Lt banks of the superior temporal sulcus | ● |  |  | ● |  |  |  |  |
| Lt inferior parietal cortex |  |  |  | ● |  |  |  |  |
| Lt middle temporal gyrus | ● | ● |  | ● |  |  |  |  |
| Lt parahippocampal gyrus | ● | ● | ● | ● | ● | ● | ● | ● |
| Lt superior temporal gyrus | ● |  |  | ● |  |  |  |  |
| Lt supramarginal gyrus | ● |  |  | ● |  |  |  |  |
| Lt frontal pole | ● | ● | ● | ● | ● | ● | ● | ● |
| Lt temporal pole | ● | ● | ● | ● | ● | ● | ● | ● |
| Lt transverse temporal cortex | ● | ● |  | ● |  |  |  |  |
| Lt insula |  |  |  |  |  |  |  | ● |
| Rt caudal middle frontal gyrus | ● | ● | ● | ● | ● | ● | ● | ● |
| Rt superior temporal gyrus | ● |  |  |  |  |  |  |  |
| Rt posterior cingulate cortex |  |  |  |  |  | ● |  |  |
| Volume | Original | +APOE ε4 | +bAge × Year interaction | Age ≥40 | Original | +APOE ε4 | +bAge × Year interaction | Age ≥40 |
| Lt caudal anterior cingulate cortex | ● | ● | ● | ● | ● |  | ● |  |
| Lt fusiform gyrus | ● | ● |  | ● |  |  |  |  |
| Lt middle temporal gyrus | ● |  |  | ● |  |  |  |  |
| Lt parahippocampal gyrus | ● | ● | ● | ● |  |  |  |  |
| Lt posterior cingulate cortex | ● | ● | ● |  |  |  |  |  |
| Lt frontal pole |  |  |  |  | ● | ● | ● | ● |
| Rt caudal anterior cingulate cortex |  |  |  |  | ● | ● | ● | ● |
| Rt caudal middle frontal gyrus | ● |  | ● | ● | ● | ● | ● | ● |
| Rt lateral orbitofrontal cortex | ● | ● | ● | ● | ● | ● | ● | ● |
| Rt posterior cingulate cortex | ● | ● |  |  | ● | ● | ● | ● |
| Rt rostral anterior cingulate cortex |  |  |  |  |  | ● |  |  |

+APOE ε4: Model including APOE ε4 status and its interaction with time. +bAge × Year interaction: Model including the interaction between baseline age and time.

**Supplementary Table S3 (A–C). Comparisons between original model and extended model.**

**Supplementary Table S3A. Thickness measure for path integration error**

| Thickness | Original vs. +APOE ε4 Status and its interaction with Year | | | | | | | | |
| --- | --- | --- | --- | --- | --- | --- | --- | --- | --- |
| ROI | AIC_base_ | AIC_extended_ | | Delta AIC | LRT p | | | LRT χ^2^ | LRT df |
| lh_bankssts | -313.1 | -314.1 | | -1.04 | 0.08 | | | 5.041 | 2 |
| lh_middletemporal | -296.6 | -293.1 | | 3.57 | 0.807 | | | 0.428 | 2 |
| lh_parahippocampal | -178.4 | -175.8 | | 2.63 | 0.503 | | | 1.373 | 2 |
| lh_superiortemporal | -331.9 | -329.8 | | 2.08 | 0.383 | | | 1.922 | 2 |
| lh_supramarginal | -380.4 | -378.4 | | 2.03 | 0.374 | | | 1.967 | 2 |
| lh_frontalpole | -153.9 | -150.5 | | 3.36 | 0.725 | | | 0.643 | 2 |
| lh_temporalpole | -112.4 | -109.2 | | 3.14 | 0.651 | | | 0.859 | 2 |
| lh_transversetemporal | -191.9 | -189.2 | | 2.63 | 0.504 | | | 1.372 | 2 |
| rh_caudalmiddlefrontal | -344.7 | -340.9 | | 3.77 | 0.893 | | | 0.227 | 2 |
| rh_superiortemporal | -368.5 | -367 | | 1.5 | 0.287 | | | 2.5 | 2 |
| Thickness | Original vs. +bAge × Year interaction | | | | | | | | |
| ROI | AIC_base_ | AIC_extended_ | | Delta AIC | | LRT p | LRT χ^2^ | | LRT df |
| lh_bankssts | -313.1 | -312 | 1.12 | | | 0.347 | 0.884 | | 1 |
| lh_middletemporal | -296.6 | -295.6 | 1.05 | | | 0.331 | 0.945 | | 1 |
| lh_parahippocampal | -178.4 | -176.8 | 1.59 | | | 0.524 | 0.406 | | 1 |
| lh_superiortemporal | -331.9 | -330.2 | 1.63 | | | 0.546 | 0.365 | | 1 |
| lh_supramarginal | -380.4 | -378.7 | 1.68 | | | 0.57 | 0.323 | | 1 |
| lh_frontalpole | -153.9 | -152.4 | 1.51 | | | 0.483 | 0.493 | | 1 |
| lh_temporalpole | -112.4 | -110.4 | 2 | | | 0.969 | 0.002 | | 1 |
| lh_transversetemporal | -191.9 | -190.4 | 1.49 | | | 0.474 | 0.513 | | 1 |
| rh_caudalmiddlefrontal | -344.7 | -344.4 | 0.25 | | | 0.186 | 1.75 | | 1 |
| rh_superiortemporal | -368.5 | -366.5 | 2 | | | 0.977 | 0.001 | | 1 |

AIC represents the Akaike Information Criterion. Delta AIC was defined as AIC_extended_ − AIC_base_, where delta AIC < 0 indicates improved overall fit after adding the biomarker terms, whereas delta AIC > 0 indicates no improvement under the information-theoretic criterion. Likelihood ratio tests (LRT) were conducted using maximum-likelihood estimation to test whether the extended models significantly improved fit relative to the base models, with corresponding p-values, likelihood ratio (χ²) statistics, and degrees of freedom (df) reported for each region of interest.

**Supplementary Table S3B. Volume measure for Path integration error**

| Volume | Original vs. +APOE ε4 Status and its interaction with Year | | | | | |
| --- | --- | --- | --- | --- | --- | --- |
| ROI | AIC_base_ | AIC_extended_ | Delta AIC | LRT p | LRT χ^2^ | LRT df |
| lh_caudalanteriorcingulate | 1792 | 1790.9 | -1.09 | 0.078 | 5.09 | 2 |
| lh_fusiform | 2160.6 | 2164.5 | 3.84 | 0.925 | 0.155 | 2 |
| lh_middletemporal | 2199.7 | 2202.5 | 2.87 | 0.569 | 1.129 | 2 |
| lh_parahippocampal | 1782 | 1778.9 | -3.11 | 0.029 | 7.109 | 2 |
| lh_posteriorcingulate | 1827.2 | 1829.5 | 2.3 | 0.427 | 1.703 | 2 |
| rh_caudalmiddlefrontal | 2060.6 | 2064.4 | 3.75 | 0.883 | 0.249 | 2 |
| rh_lateralorbitofrontal | 2096.6 | 2095.1 | -1.45 | 0.066 | 5.45 | 2 |
| rh_posteriorcingulate | 1831.7 | 1833 | 1.26 | 0.254 | 2.744 | 2 |
| Volume | Original vs. +bAge × Year interaction | | | | | |
| ROI | AIC_base_ | AIC_extended_ | Delta AIC | LRT p | LRT χ^2^ | LRT df |
| lh_caudalanteriorcingulate | 1792 | 1793.5 | 1.52 | 0.49 | 0.477 | 1 |
| lh_fusiform | 2160.6 | 2162.6 | 1.97 | 0.873 | 0.025 | 1 |
| lh_middletemporal | 2199.7 | 2201.7 | 1.99 | 0.939 | 0.006 | 1 |
| lh_parahippocampal | 1782 | 1783.6 | 1.57 | 0.513 | 0.428 | 1 |
| lh_posteriorcingulate | 1827.2 | 1828.4 | 1.22 | 0.377 | 0.78 | 1 |
| rh_caudalmiddlefrontal | 2060.6 | 2060.2 | -0.36 | 0.125 | 2.358 | 1 |
| rh_lateralorbitofrontal | 2096.6 | 2098.1 | 1.51 | 0.486 | 0.485 | 1 |
| rh_posteriorcingulate | 1831.7 | 1833.7 | 2 | 0.97 | 0.001 | 1 |

**Supplementary Table S3C. Thickness and volume measures for Angular error**

| Thickness | Original vs. +APOE ε4 Status and its interaction with Year | | | | | |
| --- | --- | --- | --- | --- | --- | --- |
| ROI | AIC_base_ | AIC_extended_ | Delta AIC | LRT p | LRT χ^2^ | LRT df |
| lh_parahippocampal | -175.4 | -172.9 | 2.49 | 0.47 | 1.509 | 2 |
| lh_frontalpole | -151.3 | -148.2 | 3.15 | 0.653 | 0.853 | 2 |
| lh_temporalpole | -112.7 | -109.7 | 3 | 0.605 | 1.004 | 2 |
| rh_caudalmiddlefrontal | -345.4 | -341.9 | 3.49 | 0.776 | 0.507 | 2 |
| rh_posteriorcingulate | -325.2 | -328.8 | -3.6 | 0.022 | 7.602 | 2 |
| Thickness | Original vs. +bAge × Year interaction | | | | | |
| ROI | AIC_base_ | AIC_extended_ | Delta AIC | LRT p | LRT χ^2^ | LRT df |
| lh_parahippocampal | -175.4 | -175.2 | 0.16 | 0.175 | 1.843 | 1 |
| lh_frontalpole | -151.3 | -149.3 | 2 | 0.975 | 0.001 | 1 |
| lh_temporalpole | -112.7 | -111 | 1.7 | 0.582 | 0.302 | 1 |
| rh_caudalmiddlefrontal | -345.4 | -343.9 | 1.49 | 0.473 | 0.515 | 1 |
| Volume | Original vs. +APOE ε4 Status and its interaction with Year | | | | | |
| ROI | AIC_base_ | AIC_extended_ | Delta AIC | LRT p | LRT χ^2^ | LRT df |
| lh_caudalanteriorcingulate | 1792.9 | 1792 | -0.85 | 0.088 | 4.854 | 2 |
| lh_frontalpole | 1588.7 | 1591.1 | 2.37 | 0.443 | 1.627 | 2 |
| rh_caudalanteriorcingulate | 1819.5 | 1819.6 | 0.04 | 0.138 | 3.957 | 2 |
| rh_caudalmiddlefrontal | 2060.7 | 2064.3 | 3.67 | 0.849 | 0.328 | 2 |
| rh_lateralorbitofrontal | 2098.1 | 2096.8 | -1.35 | 0.069 | 5.352 | 2 |
| rh_posteriorcingulate | 1830.4 | 1831 | 0.61 | 0.184 | 3.385 | 2 |
| rh_rostralanteriorcingulate | 1846.5 | 1842 | -4.5 | 0.014 | 8.501 | 2 |
| Volume | Original vs. +bAge × Year interaction | | | | | |
| ROI | AIC_base_ | AIC_extended_ | Delta AIC | LRT p | LRT χ^2^ | LRT df |
| lh_caudalanteriorcingulate | 1792.9 | 1794.9 | 2 | 0.989 | 0 | 1 |
| lh_frontalpole | 1588.7 | 1590.7 | 1.99 | 0.912 | 0.012 | 1 |
| rh_caudalanteriorcingulate | 1819.5 | 1821.4 | 1.86 | 0.706 | 0.142 | 1 |
| rh_caudalmiddlefrontal | 2060.7 | 2061.7 | 1.05 | 0.33 | 0.949 | 1 |
| rh_lateralorbitofrontal | 2098.1 | 2100.1 | 1.98 | 0.882 | 0.022 | 1 |
| rh_posteriorcingulate | 1830.4 | 1832.3 | 1.88 | 0.729 | 0.12 | 1 |

**Supplementary Table S4. ROC analysis results classifying individuals with steeper volume/thickness decline from others.**

| **ROI (Measure)** | **Percentile cutoff (steepest decline)** | **AUC (95% CI)** | **Cutoff (Error Distance)** | **Sensitivity (95% CI)** | **Specificity (95% CI)** | **Accuracy (95% CI)** | **k-fold Accuracy (95% CI)** |
| --- | --- | --- | --- | --- | --- | --- | --- |
| Left Parahippocampal (Thickness) | 10% | 0.87 (0.74–0.97) | 6.68 | 0.88 (0.67–1.00) | 0.86 (0.53–0.95) | 0.86 (0.58–0.94) | 0.84 (0.84–0.84) |
|  | 15% | 0.74 (0.52–0.92) | 6.68 | 0.73 (0.43–1.00) | 0.87 (0.77–0.97) | 0.85 (0.75–0.93) | 0.83 (0.82–0.83) |
|  | 20% | 0.64 (0.45–0.81) | 6.68 | 0.53 (0.25–0.89) | 0.86 (0.48–0.98) | 0.79 (0.55–0.89) | 0.76 (0.76–0.77) |
| Left Parahippocampal (Volume) | 10% | 0.76 (0.51–0.95) | 6.54 | 0.75 (0.42–1.00) | 0.83 (0.51–0.98) | 0.82 (0.55–0.96) | 0.79  (0.79–0.80) |
|  | 15% | 0.66 (0.45–0.85) | 6.54 | 0.55 (0.25–1.00) | 0.82 (0.38–0.98) | 0.77 (0.45–0.93) | 0.75 (0.75–0.76) |
|  | 20% | 0.70 (0.53–0.85) | 6.54 | 0.53 (0.33–1.00) | 0.84 (0.41–0.97) | 0.77 (0.51–0.89) | 0.63 (0.61–0.65) |
| Left Posterior Cingulate (Volume) | 10% | 0.75 (0.54–0.92) | 6.79 | 0.63 (0.40–1.00) | 0.86 (0.48–0.98) | 0.83 (0.52–0.94) | 0.75 (0.72–0.78) |
|  | 15% | 0.72 (0.54–0.86) | 4.29 | 0.91 (0.50–1.00) | 0.57 (0.47–0.95) | 0.62 (0.54–0.89) | 0.62 (0.61–0.62) |
|  | 20% | 0.64 (0.48–0.78) | 4.29 | 0.73 (0.31–1.00) | 0.55 (0.20–0.96) | 0.59 (0.35–0.85) | 0.54 (0.53–0.56) |
| Right Posterior Cingulate (Volume) | 10% | 0.67 (0.45–0.86) | 4.08 | 0.88 (0.33–1.00) | 0.49 (0.41–0.95) | 0.54 (0.46–0.92) | 0.63 (0.61–0.65) |
|  | 15% | 0.66 (0.46–0.85) | 6.79 | 0.55 (0.33–1.00) | 0.87 (0.46–0.95) | 0.82 (0.51–0.92) | 0.76 (0.73–0.79) |
|  | 20% | 0.61 (0.42–0.78) | 5.37 | 0.53 (0.27–0.92) | 0.75 (0.46–0.95) | 0.70 (0.52–0.86) | 0.64 (0.62–0.65) |
